# Differential Expression and Immunolocalization of Antioxidant Enzymes in *Entamoeba histolytica* Isolates during Metronidazole Stress

**DOI:** 10.1155/2014/704937

**Published:** 2014-06-12

**Authors:** Lakshmi Rani Iyer, Nishant Singh, Anil Kumar Verma, Jaishree Paul

**Affiliations:** ^1^School of Life Sciences, Jawaharlal Nehru University, New Delhi 110070, India; ^2^School of Environmental Sciences, Jawaharlal Nehru University, New Delhi 110070, India

## Abstract

*Entamoeba histolytica *infections are endemic in the Indian subcontinent. Five to eight percent of urban population residing under poor sanitary conditions suffers from *Entamoeba* infections. Metronidazole is the most widely prescribed drug used for amoebiasis. In order to understand the impact of metronidazole stress on the parasite, we evaluated the expression of two antioxidant enzymes, peroxiredoxin and FeSOD, in *Entamoeba histolytica* isolates during metronidazole stress. The results reveal that, under metronidazole stress, the mRNA expression levels of these enzymes did not undergo any significant change. Interestingly, immunolocalization studies with antibodies targeting peroxiredoxin indicate differential localization of the protein in the cell during metronidazole stress. In normal conditions, all the *Entamoeba* isolates exhibit presence of peroxiredoxin in the nucleus as well as in the membrane; however with metronidazole stress the protein localized mostly to the membrane. The change in the localization pattern was more pronounced when the cells were subjected to short term metronidazole stress compared to cells adapted to metronidazole. The protein localization to the cell membrane could be the stress response mechanism in these isolates. Colocalization pattern of peroxiredoxin with CaBp1, a cytosolic protein, revealed that the membrane and nuclear localization was specific to peroxiredoxin during metronidazole stress.

## 1. Introduction

*Entamoeba histolytica, *an enteric protozoan, is a well-established causative agent of amoebic dysentery. It has a high potential for invading and destroying human tissue. The parasite lives in the human gut in an environment of reduced oxygen pressure, and during tissue invasion it gets exposed to reactive oxygen species such as superoxide radical anions (O_2_^−^) and hydrogen peroxide (H_2_O_2_). The microaerophilic* E. histolytica *overcomes the elevated levels of oxygen and its derivatives during tissue invasion with its antioxidant enzymes. The antioxidant enzymes in* E. histolytica *are an iron containing superoxide dismutase (Fe-SOD), thioredoxin reductase (flavin reductase), and peroxiredoxin -a thiol- specific 29 kDa surface antigen, which has both peroxidase and antioxidant activity [1]. Peroxiredoxin has also been referred to as Eh 29, the 29 kDa thiol dependent peroxidase of* E. histolytica* [2]. Peroxiredoxins of* Entamoeba* have been shown to play a role in virulence by helping the parasite to survive host immune response. It plays a role in combating reactive oxygen species (ROS) and reactive nitrogen species (RNS) attack by inflammatory host cells. Peroxiredoxins can degrade hydrogen peroxide—the primary lethal oxygen derivative in* Entamoeba* effectively [3]. Studies have shown that a highly virulent strain of* Entamoeba *is less susceptible to H_2_O_2_ compared to an attenuated strain which has lost its virulence due to prolonged culturing [4, 5]. It was seen that inhibition of Eh29 gene expression led to the effective decrease in cytopathic and cytotoxic activities in* E. histolytica *trophozoites. Sizes of liver abscesses were smaller in hamsters inoculated with an Eh29 downregulated trophozoites compared to the normal HM-1 : IMSS, suggesting that peroxiredoxin of* E. histolytica *has a role in survival of trophozoites in the presence of ROS and thereby in the pathogenesis of amoebiasis [2].* E. histolytica* peroxiredoxin is induced by a high oxygen environment [6] and is also induced by Trichostatin A, a drug that increases the resistance to oxidative stress in the parasite [7].

*E. histolytica* peroxiredoxin is a galNAc lectin associated protein. It has been postulated that during host parasite interaction the lectin recruits peroxiredoxin to the host parasite surface, a mechanism by which the parasite protects itself during tissue adherence and invasion from oxidative attacks from activated host phagocytic and epithelial cells [8]. Nonvirulent* E. histolytica *was found to be more susceptible to* in vitro* oxygen challenge compared to virulent strain* E. histolytica*. In case of the virulent* E. histolytica,* resistance to oxygen challenge is due to greater ability to reduce O_2_^−^ and hydrogen peroxide as well as pyruvate ferredoxin oxidoreductase (PFOR) reactivation [9]. Peroxiredoxin expression was shown to be higher in HM-1 : IMSS, a virulent strain compared to a less virulent Rahman strain while SOD was present at a higher level in Rahman in comparison to HM-1 : IMSS [10]. It has been reported that the pathogenic* E. histolytica *contains as much as 50 times higher levels of peroxiredoxin, compared to* Entamoeba dispar*—a morphologically similar but noninvasive species [1]. Peroxiredoxin has been shown to increase by 2.1-fold in one hour high-oxygen-exposed trophozoites compared to controls [6].

Metronidazole is a synthetic 5 nitroimidazole and is used to treat infections by anaerobic protozoans like* Entamoeba* and* Giardia*. It is the single most widely prescribed drug for amoebiasis. The presence of metabolic pathways of low redox potential in* Entamoeba *and other anaerobic protozoans contributes to the selective toxicity of metronidazole to the protozoan leaving the human host unaffected. Indiscriminate use of antiamoebic drugs has led to an increase in the MIC of these drugs [11]. The enzyme Ferredoxin activates metronidazole inside the* Entamoeba *cell by reducing the nitro group of the drug into a nitroso free radical, which is cytotoxic to the cell. Thioredoxin reductase has also been reported to activate metronidazole in* Entamoeba *[12]. During the reoxidation of the active drug inside the parasite, a reactive oxygen species is generated. This reactive oxygen species is detoxified by iron containing superoxide dismutase (FeSOD) to hydrogen peroxide and oxygen. Subsequently, peroxiredoxin scavenges the hydrogen peroxide and converts it to water [13].

*In vitro *Fe-SOD activity was shown to be three times more in* Entamoeba histolytica *(HTH-56 : MUTM) strain resistant to 10 *μ*M metronidazole [14]. Laboratory induced resistance to 40 *μ*M metronidazole in* E. histolytica *(HM-1 : IMSS) was also associated with an increase in the expression of Peroxiredoxin (2.9 fold) and FeSOD (5 fold) [15].

Tazreiter et al. (2008) assessed the response of the amoeba to 50 *μ*M of metronidazole using a microarray, qRT-PCR, and two-dimensional gel electrophoresis. The results indicated only a modest increase in mRNA levels of peroxiredoxin and iron containing superoxide dismutase. The increase in mRNA expression was however not reciprocated at the protein level [16]. Schlosser et al. (2013) have reported that metronidazole treatment of* E. histolytica* reduces the activity of important oxidative stress regulatory enzymes including SOD and peroxiredoxin [17].

Since metronidazole is indiscriminately used in India, there is a possibility of development of metronidazole resistance in the* Entamoeba* isolates from India. The major aim of this work was to understand the behavior of the antioxidant enzymes during metronidazole stress in standard axenised laboratory strain HM-1 : IMSS versus clinical isolates of* Entamoeba histolytica*, from New Delhi, India, and Dhaka, Bangladesh. The expression of two genes, peroxiredoxin and Fe-SOD, involved in metronidazole inactivation and in oxidative stress response was studied in the above isolates. Immunolocalization of peroxiredoxin in the isolates was compared. Studying the behavior of these antioxidant enzymes in clinical isolates from endemic locations like Delhi and Dhaka when challenged with metronidazole will help in understanding their differential response to the drug.

## 2. Materials and Methods

### 2.1. *Entamoeba* Strains and Culture

*E. histolytica *strain HM-1 : IMSS cells were maintained and grown in TYI-S- 33 medium supplemented with 15% adult bovine serum, 1 X Diamond's vitamin mix, and antibiotics (0.3 units/mL penicillin and 0.25 mg/mL streptomycin) at 35.5°C. The cells were subcultured twice a week. Clinical isolate MS96 3382 cells from Dhaka, Bangladesh, were grown and maintained in LYI-S-2 medium supplemented as was done for TYI-S-33. MS96 3382 cells were subcultured every 48 hours. Medium was prepared as described by Clark and Diamond, 2002 [18].

### 2.2. Isolation and Maintenance of Patient Isolates of* E. histolytica* in Xenic Culture

Clinical isolate 654 was from a patient sample from Safdarjung hospital, New Delhi, while MS96 3382 (henceforth referred as MS96) was isolated from an urban slum in Dhaka. They were isolated from stool samples and maintained in Robinson's BRS medium with added* Escherichia coli *[18, 19] and subcultured thrice a week.

### 2.3. DNA Isolation and Identification of* E. histolytica*

Cells from xenic cultures were pelleted at 600 g at 4°C and stored in 70% ethanol at −20°C for DNA isolation. DNA was isolated from xenic cultures using QIA Amp DNA minikit for isolation of genomic DNA (Qiagen catalog no. 51366). Strains were identified as* E. histolytica *or* E. dispar *based on the PCR amplification using primers specific for* E. histolytica *and* E. dispar *[20].

### 2.4. HM-1 : IMSS Strain Adapted to 20 *μ*M Metronidazole

HM-1 : IMSS cells growing in mid log phase under microaerophilic conditions were exposed to low concentration of metronidazole (5 *μ*M) for 24 hours and the surviving cells were transferred to fresh medium without the drug and allowed to grow until mid-log phase. The procedure was repeated with the same concentration of drug thrice before higher drug concentrations were used following the protocol of Wassmann et al. 1999 [15]. HM-1 : IMSS cells were adapted initially to 10 *μ*M metronidazole, then to 15 *μ*M metronidazole, and finally to 20 *μ*M of metronidazole over a period of 12 months. An attempt to increase the concentration beyond was unsuccessful as the surviving cells could not proliferate. These cells, adapted to 20 *μ*M metronidazole, were henceforth referred to as 20 *μ*M A and were subsequently maintained in 20 *μ*M metronidazole. The percent cell survival in metronidazole after 72 hours was estimated using trypan blue. Cell counting was done using a haemocytometer.

### 2.5. Short Term Metronidazole Stress to Xenic and Axenic Cells

In case of axenic cells (HM-1 : IMSS) and MS 96 (AX), the cells were grown till the log phase in 2 × 50 mL flasks in TYIS33 and LYI S-3 medium, respectively. The medium was then decanted and fresh medium with 20 *μ*M metronidazole was added. The cells were then incubated for 24 hours with the drug at 35.5°C. These metronidazole treated cells will be henceforth referred to as 20 *μ*M S and MS96 (AX) 20 *μ*M, respectively. The treated cells were harvested for RNA isolation and immunofluorescence studies. Trizol reagent was added to the cell pellet and mixed thoroughly by pipetting and then pellet was stored at −80°C for RNA isolation. For immunofluorescence microscopy, the harvested cells were washed in PBS buffer and fixed in cold methanol for at least 20 minutes at −20°C.

In case of xenic culture eight tubes (5 mL Bijou bottles) each of cells MS96 (X) and 654 were grown in Robinson's medium till log phase (24 hours); then they were harvested by centrifugation at 600 g for 5 minutes at 4°C and cells (approximately 50,000 cells per bottle) were resuspended in 3 mL fresh BRS having concentration of 25 *μ*M of metronidazole in case of MS96 (X) and 50 *μ*M in case of clinical isolate 654. This was overlaid on a new saline agar slant in 5 mL Bijou bottles. The medium was completed and bottles were incubated at 37°C for 24 hours and then harvested by centrifuging at 600 g for 5 minutes in RNAase free falcon tubes (50 mL). The supernatant was carefully decanted. The harvested cells were stored for RNA isolation and immunofluorescence microscopy as described in case of axenic cells. Cell survival after metronidazole treatment was counted using haemocytometer. The clinical isolates treated with metronidazole were termed as 654 (50 *μ*M) and MS96 (X) 25 *μ*M, respectively.

### 2.6. Expression Level of Antioxidant Enzymes Peroxiredoxin and Superoxide Dismutase by Semiquantitative RT-PCR

#### 2.6.1. Isolation of RNA

Total RNA was isolated from normal and treated* E. histolytica *trophozoites incubated for time periods ranging from 0 h to 24 h using Trizol reagent (Invitrogen) following the manufacturer's protocol. The isolated RNA was treated with DNase (Roche) according to the manufacturer's protocol.

#### 2.6.2. Semiquantitative RT-PCR

Reverse transcription was performed using 5 *μ*g of DNase treated RNA from normal cells and stress induced* E. histolytica *cells using a random Hexamer from Promega and MMLV RT enzyme following manufacturers protocol.

The synthesized cDNA was amplified using set of peroxiredoxin and FeSOD forward and reverse primers. The sequence of* Entamoeba* peroxiredoxin has been cloned and reported earlier by Torian et al. (1990) and Bruchhaus and Tannich (1993) [21, 22]. Primer sequences and accession numbers of peroxiredoxin and FeSOD are as shown in Table 1. The isoforms of entamoeba peroxiredoxins to which our peroxiredoxin primers set bind were checked using Eupath Db (http://amoebadb.org/amoeba/). The primers selected for this study amplified eleven isoforms of peroxiredoxin. 18S rRNA was used for normalization. PCR amplification of the target gene and 18S rRNA was carried out in the same tube simultaneously. In all the reactions, initial denaturation was carried out at 94°C for 5 min, targeted genes were amplified by 30 amplification cycles, annealing was done for 1 minute at 50°C for peroxiredoxin and 18S rRNA at 48°C for FeSOD, and an extension was done at 72°C for 1 min followed by a final extension at 72°C for 5 min. The products were run on 1.2% agarose gel, stained with ethidium bromide, and finally documented and quantified using the Alpha Imager Gel Documentation System. In case of axenic cultures a nontemplate control was used while in case of xenic cultures, cDNA was prepared from total RNA, isolated from uninoculated xenic culture medium containing only bacteria, and was used as a blank.

#### 2.6.3. Spot Densitometry

The bands obtained after electrophoresis were evaluated using spot densitometry with Alpha Ease FC software. Densitometric value of peroxiredoxin and Fe-SOD band area of each cell line were expressed as percent of 18S rRNA band density. The experiment was repeated with at least three sets of RNA on all* Entamoeba *isolates studied.

#### 2.6.4. Statistical Analysis

Mean, standard deviation, and paired* t-*test for the control set and treated groups were carried out in case of normal and metronidazole stress conditions. ANOVA test was employed for comparing the expression of antioxidant enzymes between different isolates.

### 2.7. Immunofluorescence Microscopy

Cells were harvested in the log phase and then washed with PBS buffer, fixed in cold methanol for 20 m at (−20°C), washed twice with PBS, and permeabilized with 0.1% triton-X-100 for 5 min at room temperature, washed, and blocked with 3% BSA at R.T for 60 min. After blocking, cells were washed thrice with PBS and incubated for 45 min at RT with monoclonal antiperoxiredoxin antibody generated in mouse at 1 : 250 dilution, washed with PBS, and incubated for 30 min with anti-mouse secondary antibody tagged with Alexa-448 in 1 : 500 dilution along with nuclear strain Hoechst 33342 (2.5 *μ*g/mL).

Colocalization of peroxiredoxin with another cytoplasmic protein CaBp1 was also performed [23]. Blocked cells were incubated with antiperoxiredoxin antibody generated in mouse together with a monoclonal anti-CaBp1 antibody generated in rabbits (1 : 250). The cells were washed with PBS and incubated for 30 m with anti-mouse secondary antibody tagged with Alexa-488, and anti-rabbit secondary antibody tagged with Alexa 555 in 1 : 500 dilution and Hoechst 33342 (2.5 *μ*g/mL). Colocalization studies with fibrillarin using polyclonal anti-rabbit anti-fibrillarin antibody were also used to confirm the nucleolar localization of peroxiredoxin in the cells according to the method of Jhingan et al. 2009 [24]. Trophozoites were then washed thrice with PBS and mounted on glass slide in 25% glycerol with antibleach p-phenylenediamine (Sigma). All steps were done in 1.5 mL microfuge tubes by pelleting at 4°C, 500 g for 5 min. Phase contrast and fluorescent images were taken using a Zeiss Axio Imager M1 microscope (Germany) with a 100x magnification.

## 3. Results

### 3.1. Effect of Metronidazole on the Growth of Different Strains of* E. histolytica*

**Figure 1:**
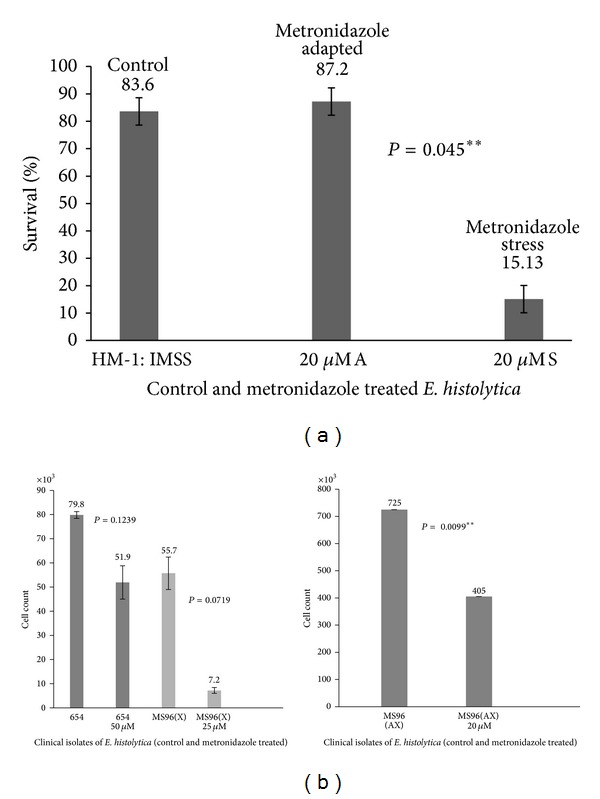
(a) Percent cell survival in HM-1 : IMSS after treatment with metronidazole. Percent cell survival after treatment with 20 *μ*M metronidazole for 72 hours was estimated using trypan blue. Cell survival during metronidazole shock (20 *μ*M S) was significantly low compared to adapted cells (20 *μ*M A) and untreated HM-1 : IMSS cells. *P* = 0.045. (b) Cell count in clinical isolates of* E. histolytica* after metronidazole treatment. Cell counts in clinical isolates were carried out 24 h after treatment with metronidazole. Each pair of columns shows the untreated and treated cells of each strain.* P* values were calculated using paired* t*-test.

Cell survival of HM-1 : IMSS cells and 20 *μ*M A cells (adapted to 20 *μ*M metronidazole) was assessed by counting percent cell survival after 72 h. Cell survival was significantly higher both in 20 *μ*M A cells as well as untreated HM-1 : IMSS cells in comparison to the HM-1 : IMSS cells given a 20 *μ*M metronidazole shock for 72 h (20 *μ*M S) (Figure 1(a)). This shows that the 20 *μ*M A cells were able to survive and multiply constantly in the presence of the 20 *μ*M metronidazole while the 20 *μ*M S cells could not. The growth of clinical isolates in the presence of metronidazole was assessed by cell count after 24 hours of treatment.The concentration of metronidazole chosen for each clinical isolate was based on plate tests done to calculate MIC according to the method of Upcroft and Upcroft, 2001 [25]. MIC values were found to be 20 *μ*M, 25 *μ*M, and 50 *μ*M for MS96 (AX), MS96 (X), and 654, respectively. Cell counts in the treated population were lower compared to untreated cells in all the three clinical isolates (Figure 1(b)). In case of axenic isolate MS96 (AX) the reduction in cell count was significant (*P* = 0.0099).

### 3.2. Metronidazole Stress Responses in HM-1 : IMSS Cells by RT PCR

**Figure 2:**
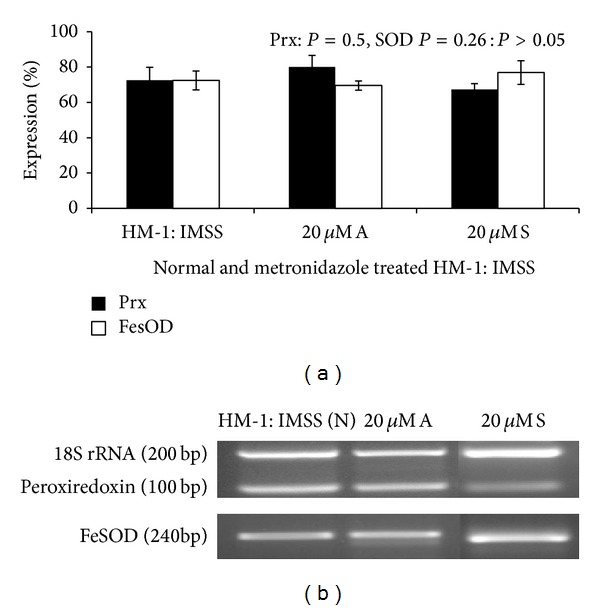
(a) Expression of peroxiredoxin and FeSOD in HM-1 : IMSS cells during Metronidazole stress. The densitometric data from semiquantitative RT-PCR analysis of peroxiredoxin (Prx) and FeSOD during metronidazole stress in HM-1 : IMSS are represented graphically. Data are mean ± SD of three independent experiments. 18S rRNA PCR was used as an internal control. Densitometric values were expressed as % after normalizing with 18S rRNA. *P* > 0.05. (b) Representative gel images.

The expression of both the antioxidant enzymes peroxiredoxin and SOD did not change significantly either in 20 *μ*M S or 20 *μ*M A when compared to untreated cells as measured by RT PCR (*P* > 0.05) (Figures 2(a) and 2(b)).

### 3.3. Immunolocalization of Peroxiredoxin in HM-1 : IMSS Cells

**Figure 3:**
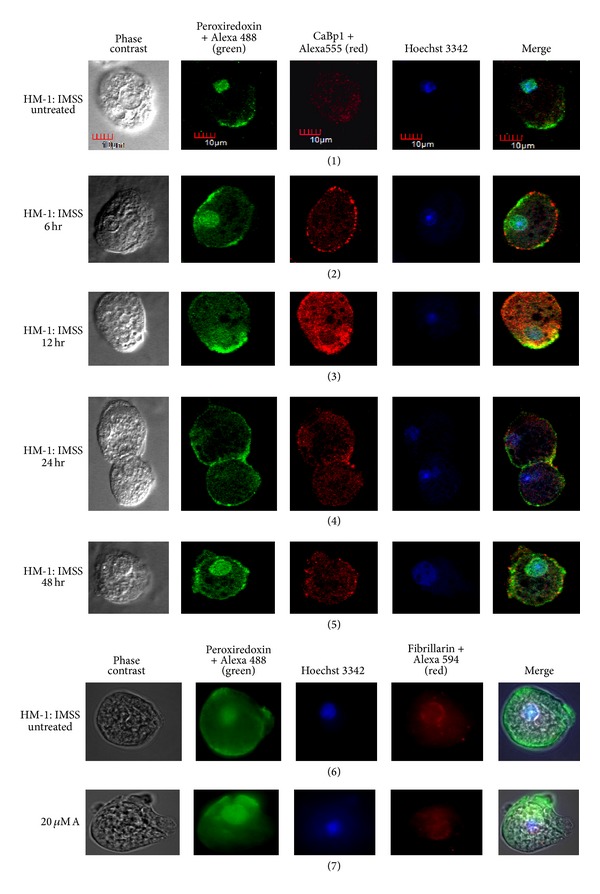
Immunolocalization of peroxiredoxin in* E. histolytica *strain HM-1 : IMSS during metronidazole stress. The images from left to right in each panel show phase contrast, peroxiredoxin stained by Alexa 488 (green), CaBp-1 stained by Alexa-555, Hoechst 3342 for nuclear staining, and the merged image. Peroxiredoxin was present in the membrane and the nucleus in the untreated cells (panel (1)), during short term metronidazole shock the peroxiredoxin localized to the membrane with loss of nuclear staining within 24 hours (panels (2), (3), and (4)) and then reverted back to the normal position after 48 hours (panel (5)). CaBp1 localized to the cytoplasm. Panels (6) and (7) show untreated cells and cells adapted to metronidazole (20 *μ*M A), respectively. Peroxiredoxin localized to nucleus and membrane in both cases. The fourth panel from left shows fibrillarin stained with Alexa 594 to mark the nucleolus (panels (6) and (7)).

For studying the localization of peroxiredoxin, we took an average of forty cells per group. The enzyme localized principally to two cellular compartments (nucleus and membrane) in more than 50% of untreated cells. In order to confirm the nuclear localization of peroxiredoxin, we used two stains, one specific for nucleus (Hoechst) and anti-fibrillarin antibody to stain the nucleolus. In 15 to 20% cells the peroxiredoxin protein was localized to the membrane and in less than 5% cells to the nucleus only. In order to see whether membrane localization was characteristic of all cytoplasmic proteins, colocalization of CaBp1 along with peroxiredoxin was studied in these cells. It was seen that CaBp1 localized in the cytoplasm in 70% cells while peroxiredoxin localized to nucleus and membrane. In 30% cells CaBp1 showed membrane localization and it was exclusively at the site of phagocytic cup. Peroxiredoxin was seen to localize to specific pockets of the membrane rather than the whole membrane in 25% of the cells (Figure 3, panel (1)).

Peroxiredoxin localization in cells adapted to grow in metronidazole (20 *μ*M A) was similar to that of untreated cells with both nuclear and membrane localization (Figure 3, panels (6) and (7)). Nuclear localization of peroxiredoxin protein was further confirmed by the nucleolus staining with fibrillarin.

During (20 *μ*M S) metronidazole shock given over a period of 6 hours, 80% of the cells showed membrane and nuclear localization of peroxiredoxin and 20% cells showed only membrane localization while CaBp1 localized to the cytoplasm only even during shock (Figure 3, panel (2)). However, after 12 h of metronidazole shock, 70% cells showed membrane localization and 15% showed membrane and nuclear localization while 15% showed only nuclear localization. CaBp1 was localized in the cytoplasm and in some cells only at the site of phagocytosis (Figure 3, panel (3)). After 24 h, in 87% of cells the peroxiredoxin localized only to the membrane without any nuclear localization while the rest of the cells showed both membrane and nuclear localization (Figure 3, panel (4)). After 48 h of shock, the localization of peroxiredoxin in cells tends to revert back to normal condition showing both membrane and nuclear localization together (Figure 3, panel (5)). Our observation shows that the introduction of metronidazole triggered a flux of peroxiredoxin to the membrane from the nucleus within 24 h of stress which reversed after a period of 48 h.

The localization to the nucleus and membrane was specific to peroxiredoxin because on colocalization with CaBp1 in standard HM-1 : IMSS cells, peroxiredoxin localized mostly to the membrane and to the nucleus in contrast to CaBp1 localization, which was cytoplasmic and localized to the membrane (only during phagocytosis) but never to the nucleus as reported earlier [23].

### 3.4. Expression of Peroxiredoxin and FeSOD in Clinical Isolates

**Figure 4:**
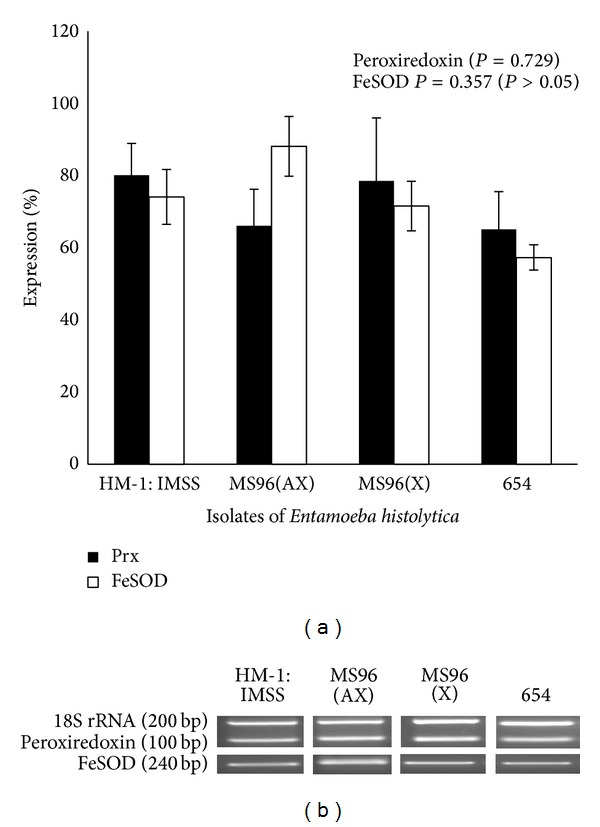
(a) Expression of peroxiredoxin and FeSOD in different isolates of* E. histolytica.* Graphical representation of densitometric data from semiquantitative RT-PCR analysis of Peroxiredoxin and FeSOD in* E. histolytica *trophozoites. No significant difference was seen in the expression levels of the two enzymes between the different isolates (*P* > 0.05). (b) Representative gel images.

In untreated conditions, peroxiredoxin and FeSOD expression levels did not show any significant change either in the clinical isolates maintained in xenic conditions or MS96 (AX) maintained in axenic conditions, compared to HM-1 : IMSS. One-way ANOVA was used to analyze the data (Figure 4).

**Figure 5:**
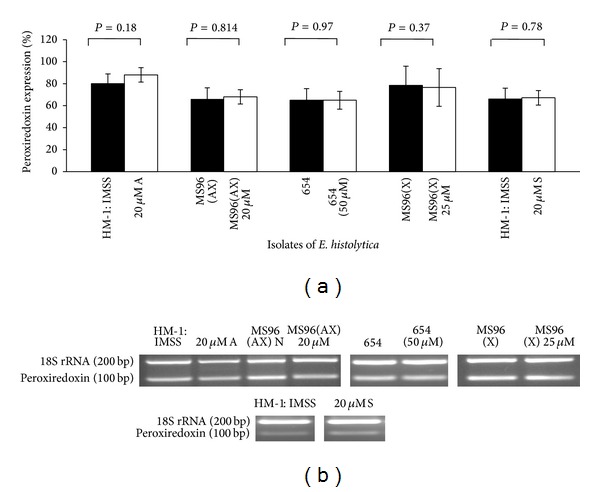
(a) Expression of peroxiredoxin in different isolates of* E. histolytica* during metronidazole stress. Graphical representation of densitometric data from semiquantitative RT-PCR analysis of peroxiredoxin in different isolates of* E. histolytica* in conditions of metronidazole stress. RT-PCR details as described in Figure 2. Each pair of columns shows the untreated and treated cells of isolates. Data are mean ± S.E for at least three independent experiments. *P* > 0.05. (b) Representative gel images.

**Figure 6:**
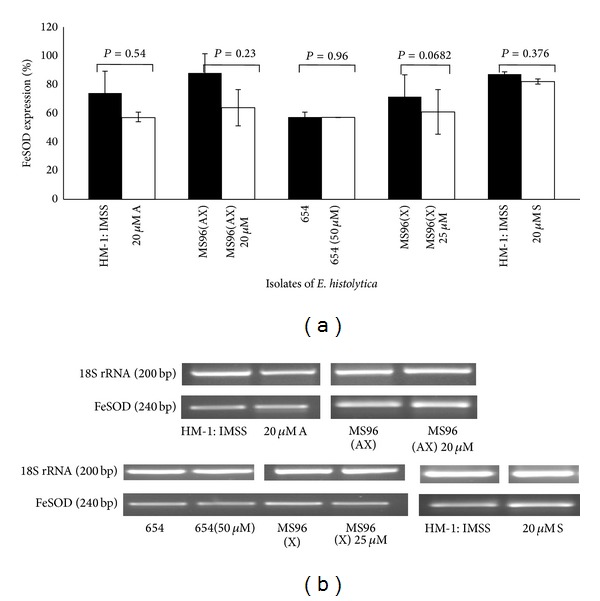
(a) Expression of FeSOD in different isolates of* E. histolytica* during metronidazole stress. Graphical representation of densitometric data from semiquantitative RT-PCR analysis of FeSOD in different isolates of* E. histolytica* in conditions of metronidazole stress. RT-PCR details are as described in Figure 2. Each pair of columns shows the untreated and treated cells of a strain. Data are mean ± S.E for at least three independent experiments. *P* > 0.05. (b) Representative gel images.

When clinical isolates were subjected to different concentrations of metronidazole shock (20, 25, and 50 *μ*m for MS96 (AX), MS96 (X), and 654, resp.) no significant changes were observed in the expression level. However MS96 (X) exhibited consistent decrease in expression of FeSOD gene but did not attain a significant value when data was analyzed using paired* t-*test (Figures 5 and 6).

### 3.5. Immunolocalization of Peroxiredoxin in Clinical Isolates

**Figure 7:**
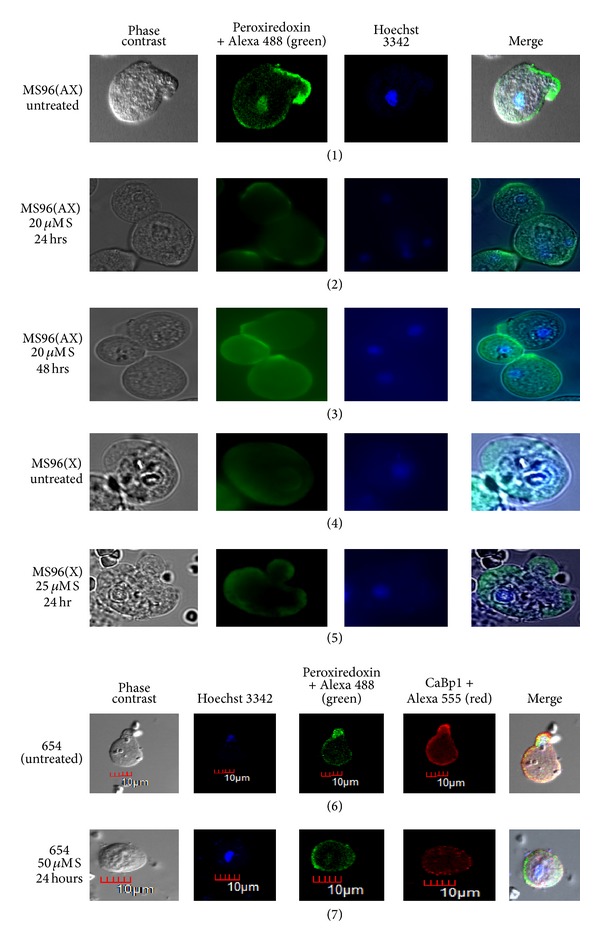
Immunolocalization of peroxiredoxin in clinical isolates of* E. histolytica* given short term metronidazole stress. The images from left to right in each panel show phase contrast, peroxiredoxin stained by Alexa 488, Hoechst 3342 for nuclear staining, and the merged image. In normal conditions Peroxiredoxin localized to the membrane and to the nucleus in axenic clinical isolate MS96 (AX) (panel (1)). After 24 and 48 hours of metronidazole stress peroxiredoxin localized to the membrane with loss of nuclear staining (panels (2) and (3)). Xenic isolates MS96 (X) and 654 showed loss of nuclear staining with metronidazole shock (panels (4) to (7)). Panels (6) and (7) show cytoplasmic localization of CaBp1 during the stress.

In axenically grown clinical isolate MS96, peroxiredoxin localized to pockets of the membrane and also to the nucleus in untreated cells (Figure 7, panel (1)). When the cells were given 20 *μ*M metronidazole shock for 24 h, peroxiredoxin localized to the periphery of cytoplasmic membrane with loss of nuclear staining in more than 80% of the cells (Figure 7, panel (2)). After 48 h of shock, all the cells reverted back to normal condition showing both nuclear and membrane localization of peroxiredoxin (Figure 7, panel (3)).

In case of xenic isolate MS96 and 654 the protein localized both to the nucleus and membrane in untreated cells but during metronidazole shock, it localized only to the membrane (Figure 7, panels (4) to (7)). Our colocalization study using CaBp1 in xenic isolate 654 confirmed similar results as observed in the axenic isolates (Figure 7, panels (6) and (7)).

## 4. Discussion

Our results have shown that the expression of both the antioxidant genes did not change significantly during metronidazole stress conditions either in HM-1 : IMSS strain or in clinical isolates. The reports dealing with the changes in the expression of antioxidant enzymes during metronidazole stress are not conclusive [14, 15]. According to the recent report by Schlosser et al. (2013) a reduction in Peroxiredoxin and SOD enzyme activity is explained to be linked to the Thioredoxin reductase/Thioredoxin system. Reduced thioredoxin donates the electron required for peroxiredoxin to detoxify H_2_O_2_. It was explained that adduct formation between thioredoxin reductase (TrxR) and reduced metronidazole affects the thioredoxin reductase activity of TrxR leading to a decreased activity of peroxiredoxin [17]. Other reports also reveal similar behavior of antioxidant enzymes (FeSOD and peroxiredoxin) in* E. histolytica* which were not modulated in different stress conditions [26, 27].

Earlier studies on the localization of peroxiredoxin in* Entamoeba *revealed membrane localization of peroxiredoxin mostly in formalin fixed cells that were pretreated with the antibody against peroxiredoxin [1, 21]. Tachibana et al.1990 and Cheng et al. 2004 [28, 29] demonstrated that this protein was localized both in the nucleus and cytoplasm using a polyclonal antibody. However, our results demonstrate nuclear and membrane localization of peroxiredoxin using monoclonal antibodies against peroxiredoxin on* Entamoeba histolytica *cells fixed in methanol and permeabilized by Triton X100. The nuclear localization of peroxiredoxin was confirmed by Hoechst and Fibrillarin staining of the nucleus. Localization of peroxiredoxin in the nucleus along with membrane in untreated cells is reasonable if the protection of DNA from oxidative stress is mediated by peroxiredoxin.

Our results however clearly reveal a substantial change in localization pattern of peroxiredoxin during metronidazole stress indicating its recruitment to the surface. This response was observed both in standard laboratory strain HM-1 : IMSS as well as in clinical isolates. The localization of CaBp1, another cytoplasmic protein, was exclusively in the cytoplasm and never to the nucleus unlike peroxiredoxin. CaBp1 however localized to the membrane only during phagocytosis as reported earlier [23]. Thus it can be concluded that the protein peroxiredoxin behaved differently from this cytosolic protein CaBp1. During metronidazole stress, peroxiredoxin was localized mostly to specific pockets of the membrane; however, these pockets are still not defined at present.

When we adapted the HM-1 : IMSS cells to 20 *μ*M metronidazole for a period of one year, we observed similar behavior of the cells as untreated HM-1 : IMSS showing the localization of peroxiredoxin both in the nucleus and the membrane. Thus we can infer that the relocalization of the enzyme to the membrane is an immediate and not a long term response.

Our results revealed that the Indian isolate could tolerate higher concentration of the drug compared to standard strains. Our study further concentrated on the expression of the antioxidant enzymes in the clinical isolates of* Entamoeba* when exposed to short term or long term metronidazole stress. Changes in the localization pattern of the antioxidant enzyme peroxiredoxin observed in our isolates during metronidazole shock indicated the protective role of this antioxidant enzyme towards the drug. This supported our hypothesis about how the clinical isolates show differential tolerance to the drug encountered in the host. We further conclude that the change in localization of the antioxidant enzyme is however not reciprocated by an increased mRNA expression of the protein. To our knowledge, this is for the first time, the expression of antioxidant genes, peroxiredoxin, and super oxide dismutase and the immunolocalization pattern of peroxiredoxin has been demonstrated in clinical isolates of* Entamoeba histolytica*.

## Figures and Tables

**Table 1:** Description of primers used for RT_PCR.

Enzyme	Primer composition	Tm	Amplicon size
*Eh* peroxiredoxin (Prx)	F 5′ AAA TCA ATT GTG AAG TTA TTG G 3′ R 5′ TCC TAC TCC TCC TTT ACT TTT A 3′	53.6°C 56.8°C	100 bp

Fe SOD Accession number (XM_643735.2)	F 5′ ACA ATT ACC TTA TGC TTA TAA 3′ R 5′ TCC ACA TCC ACA CAT ACA AT 3′	52°C 54°C	240 bp

*Entamoeba histolytica* 18S ribosomal RNA gene	F 5′ TCA GCC TTG TGA CCA TAC TC 3′ R 5′ AAG ACG ATC AGA TAC CGT CG 3′	61.7°C 68.9°C	200 bp
